# Identification of driver genes in lupus nephritis based on comprehensive bioinformatics and machine learning

**DOI:** 10.3389/fimmu.2023.1288699

**Published:** 2023-12-07

**Authors:** Zheng Wang, Danni Hu, Guangchang Pei, Rui Zeng, Ying Yao

**Affiliations:** ^1^ Division of Nephrology, Tongji Hospital, Tongji Medical College, Huazhong University of Science and Technology, Wuhan, China; ^2^ Key Laboratory of Organ Transplantation, Ministry of Education, Wuhan, China; ^3^ NHC Key Laboratory of Organ Transplantation, Chinese Academy of Medical Sciences, Wuhan, China; ^4^ Key Laboratory of Organ Transplantation, Chinese Academy of Medical Sciences, Wuhan, China; ^5^ Department of Nutrition, Tongji Hospital, Tongji Medical College, Huazhong University of Science and Technology, Wuhan, China

**Keywords:** Lupus nephritis, bioinformatics, machine learning, immune infiltration, WGCNA

## Abstract

**Background:**

Lupus nephritis (LN) is a common and severe glomerulonephritis that often occurs as an organ manifestation of systemic lupus erythematosus (SLE). However, the complex pathological mechanisms associated with LN have hindered the progress of targeted therapies.

**Methods:**

We analyzed glomerular tissues from 133 patients with LN and 51 normal controls using data obtained from the GEO database. Differentially expressed genes (DEGs) were identified and subjected to enrichment analysis. Weighted gene co-expression network analysis (WGCNA) was utilized to identify key gene modules. The least absolute shrinkage and selection operator (LASSO) and random forest were used to identify hub genes. We also analyzed immune cell infiltration using CIBERSORT. Additionally, we investigated the relationships between hub genes and clinicopathological features, as well as examined the distribution and expression of hub genes in the kidney.

**Results:**

A total of 270 DEGs were identified in LN. Using weighted gene co-expression network analysis (WGCNA), we clustered these DEGs into 14 modules. Among them, the turquoise module displayed a significant correlation with LN (cor=0.88, p<0.0001). Machine learning techniques identified four hub genes, namely CD53 (AUC=0.995), TGFBI (AUC=0.997), MS4A6A (AUC=0.994), and HERC6 (AUC=0.999), which are involved in inflammation response and immune activation. CIBERSORT analysis suggested that these hub genes may contribute to immune cell infiltration. Furthermore, these hub genes exhibited strong correlations with the classification, renal function, and proteinuria of LN. Interestingly, the highest hub gene expression score was observed in macrophages.

**Conclusion:**

CD53, TGFBI, MS4A6A, and HERC6 have emerged as promising candidate driver genes for LN. These hub genes hold the potential to offer valuable insights into the molecular diagnosis and treatment of LN.

## Introduction

Systemic lupus erythematosus (SLE) is a complex autoimmune disorder triggered by a variety of endogenous antigens (1, 2). Lupus nephritis (LN) is a common and severe immune complex glomerulonephritis that occurs as a target-organ manifestation of SLE. It is characterized by symptoms, including hematuria, proteinuria, and impaired renal function (3, 4). LN often appears within five years of SLE diagnosis, affecting approximately 50% of patients (4, 5). Despite progress in understanding the pathogenesis of LN, treatment advancements have been limited (6). The incidence of kidney failure remains unacceptably high, with about one-third of individuals with severe LN at risk of developing end-stage renal disease (ESRD) within a decade (7–9).

In individuals with SLE, the immune system produces autoantibodies and immune complexes that gradually accumulate within the renal glomeruli (10–12). This accumulation triggers an inflammatory response, resulting in glomerular damage and dysfunction. Commonly observed histopathological abnormalities in the glomeruli consist of immune complex deposition, increased proliferation of mesangial and endothelial cells, inflammatory cell infiltration, cellular crescent formation, and injury to the glomerular basement membrane (GBM). Therefore, the renal glomerulus plays a crucial role in the onset and progression of LN (13, 14).

Recent research has provided insights into the involvement of susceptibility genes in LN, disrupting immune tolerance and contributing to the disease’s development. These genes amplify innate immune signaling pathways and promote lymphocyte activation, ultimately resulting in renal damage (15–19). Autoreactive leukocytes, immune complexes, complement proteins, and various inflammatory mediators also play significant roles in LN’s development (20, 21). Understanding the molecular mechanisms underlying LN could lead to the development of more effective treatment strategies.

The infiltration of immune cells plays a crucial role in the development and progression of kidney diseases. Targeting specific immune cell populations or manipulating their functions could alleviate inflammation, decrease tissue damage, and improve the prognosis for patients with kidney diseases (22–24).

The emergence of gene microarray technology and high-throughput techniques has made bioinformatics methods essential for efficiently identifying DEGs (25–28). In recent years, machine learning (ML) has found wide application in addressing complex problems in the biomedical field. ML’s capabilities in analyzing large datasets and uncovering valuable relationships make it an effective tool for elucidating patterns and providing explanations (29–31). Integrating bioinformatics analysis with ML offers prospective opportunities to enhance the accuracy, reliability, and predictability of disease diagnosis. In this study, we utilized bioinformatics methods to acquire gene expression matrices from glomerular tissues of LN patients obtained from the GEO database. We performed differential expression analysis, enrichment analysis, and investigated candidate hub genes using weighted gene co-expression network analysis (WGCNA). Two machine learning algorithms, LASSO regression and random forest, were employed to identify hub genes associated with LN. Glomerular immune infiltration in LN and normal controls was quantified using the CIBERSORT algorithm based on gene expression profiles. Furthermore, associations between hub genes and immune infiltration, as well as clinical and pathological features in LN, were examined. The distribution and expression patterns of these hub genes were also identified. The primary objective of these analyses is to offer novel insights that can contribute to the prevention and treatment of LN.

## Materials and methods

### Searching and downloading of microarray data

The microarray datasets of LN were obtained from the National Center for Biotechnology Information Gene Expression Omnibus (GEO) database (http://www.ncbi.nih.gov/geo/) by utilizing the keyword “lupus nephritis” as the search criteria. The datasets were chosen based on the criteria that: (1) the study type is expression profiling by array, (2) the attribute name is glomerular tissue, and (3) the organisms are Homo sapiens. Four gene expression datasets (GSE99339, GSE104948, GSE127797, and GSE32591) were identified as eligible. These datasets comprise a total of 133 patients with LN and 51 normal controls.

### Data pre-processing

Initially, the probe matrix was transformed into a gene matrix using the GEOquery package, and the probe annotation file was employed in the process. If multiple probes were associated with the same gene, the gene’s expression value was determined by calculating the average value across the probes. Secondly, since these four datasets were obtained from different platforms and exhibited batch effects, the sva package was employed to mitigate batch effects across the various platforms.

### Identification of differentially expressed genes

The limma package was utilized for the analysis of DEGs between patients with LN and normal controls. The criterion used for selecting DEGs were a p-value < 0.05 and a fold change (FC) > 1 in absolute value.

### Enrichment analysis

The biological processes of Gene Ontology and Kyoto Encyclopedia of Genes and Genomes of DEGs were enriched using the clusterProfiler package in R. Pathways were considered significantly enriched if the adjusted p-value was less than 0.05.

### Construction of the Weighted Gene Co-expression Network Analysis network

To identify the co-expression network and select genes from different clusters, we used the top 5000 standard variance genes to construct weighted gene co-expression network using the WGCNA package (32). The selection of a soft threshold power (β) was determined by applying the pickSoftThreshold function and adhering to the scale-free topology criterion. Subsequently, gene co-expression modules were identified through the utilization of the one-step network construction method. Each module consisted of a minimum of 30 genes, while any remaining ungrouped genes were assigned to the grey module. We calculated correlation coefficients between modules and phenotypes to identify modules that were closely associated with LN. Additionally, the connection between gene modules and LN patients was evaluated by assessing the values of gene significance (GS) and module membership (MM).

### Reactome pathway analysis among different modules

The Reactome pathway analysis was conducted on the genes from different modules using the clusterProfiler package in R. The “compareCluster” function was used with the parameter “fun” set to “enrichPathway” for the selection of enriched pathways. Significantly enriched pathways were defined as those with an adjusted p-value below 0.05.

### Construction training group and validation group

The training and validation groups were divided utilizing the caret package, a widely-used tool for statistics and machine learning. The package provides a convenient and efficient approach for training and evaluating models. The data splitting strategy involved randomly assigning samples to the training and validation groups in a ratio of 7:3. The createDataPartition function was utilized to conduct random sampling, ensuring that the samples were representative of the overall distribution in both groups.

### LASSO regression screening of hub genes

LASSO regression is a commonly-used machine learning algorithm employed to fit generalized linear models. It is acknowledged for its capability to simultaneously perform variable selection and complexity regularization (33). LASSO regression utilizes the parameter λ to adjust the complexity level. Increasing the value of λ imposes a higher penalty on linear models with a large number of variables. This results in a reduced number of selected genes, leading to a more concise and representative set of key genes in the outcome. The glmnet package in R was used to conduct the LASSO regression analysis of candidate hub genes in our study. The optimal value of λ was determined through ten-fold cross-validation, selecting the value that resulted in the minimum criterion.

### Random forest model screening of hub genes

The random forest model is a machine learning approach that uses multiple independent decision trees to predict classification or regression (34). In this study, we utilized the R package “randomforest” to construct our random forest model. To determine the optimal number of variables, we calculated the average error rate for candidate hub genes. We then assessed the error rate for tree numbers ranging from one to 500, and selected the number of trees with the lowest error rate. Once the parameters were determined, we built the random forest tree model. Lastly, we identified the feature importance scores for each candidate hub gene and selected the genes with an importance value greater than 0.25.

### Diagnostic value of hub genes in LN

In order to test the accuracy of the hub genes screened by machine learning, the ROC curves were generated between the LN patients and the normal controls in training group. The greater the area under curve (AUC), the higher the accuracy of the gene as a hub gene in LN. In the same method, its effectiveness was further verified in the validation group.

### Gene set enrichment analysis

To investigate the association between hub genes and signaling pathways, we divided the LN group into two subgroups using the median value of hub gene expression. Subsequently, we performed gene set enrichment analysis (GSEA) on each subgroup, with a significance level set at adjusted p-value < 0.05.

### Identification of immune cell infiltration

The CIBERSORT algorithm, which utilizes linear support vector regression (SVR), is a widely used and trustworthy machine learning method for deconvoluting the expression matrix of 22 human immune cell subtypes (35). In this study, we employed the CIBERSORT algorithm to determine the relative proportions of different immune cells in the LN samples and normal controls. To ensure accurate results, we performed 1,000 calculations.

### Correlation analysis between hub genes and infiltrating immune cells

Correlation analysis between hub genes and immune cells was performed using Spearman correlation coefficient.

### Correlation of hub genes with clinicopathological features

Based on the Nephroseq database(https://nephroseq.org/), the correlations of hub genes with different pathological calcification, renal function and proteinuria were analyzed in patients with lupus nephritis.

### Identification and distribution analysis of hub genes in the kidney

Based on the raw single-cell RNA-seq data deposited in dbGAP (accession code phs001457.v1.p1), and the processed data available for viewing using an interactive browser at https://immunogenomics.io/ampsle/ and https://singlecell.broadinstitute.org/single_cell/study/SCP279/amp-phase-1, the distribution and expression of hub genes were calculated (22).

### Ethics statement

The present study solely relied on pre-existing data obtained from publicly accessible sources, no specific ethical considerations such as informed consent, confidentiality, or participant privacy were applicable or involved in this research.

### Statistical analysis

**Figure 1:**
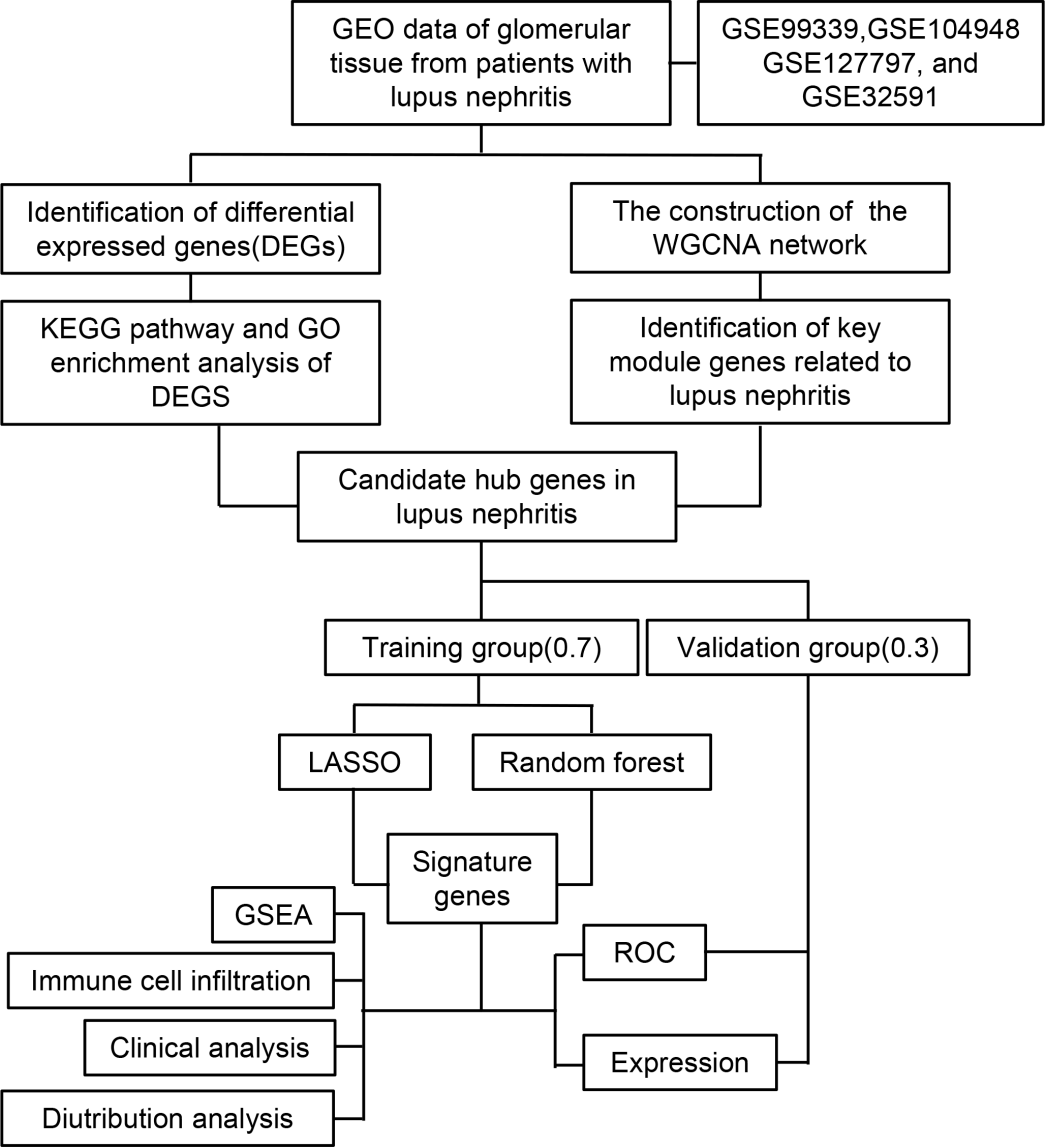
Flow chart of the research study.

Statistical analysis of the data from this study was performed using R (Version 4.2.2). A t-test was conducted for continuous variables between two groups, assuming they followed a normal distribution. To investigate the correlation between gene expression and immune cell fraction, the Spearman rank correlation test was employed. The statistical significance level was set at p-value < 0.05. The flow chart of this research was shown in **Figure 1**.

## Results

### Identification of DEGs between LN and normal controls

The microarray datasets GSE99339, GSE104948, GSE127797, and GSE32591 were obtained from the GEO database. These datasets comprise 133 patients with LN and 51 normal controls. After conducting quality control procedures on these four datasets, it was observed that the expression levels remained consistent among the samples. This consistency suggests that any potential batch effects have been successfully mitigated, thereby facilitating subsequent research (**Figures S1A-F**).

**Figure 2:**
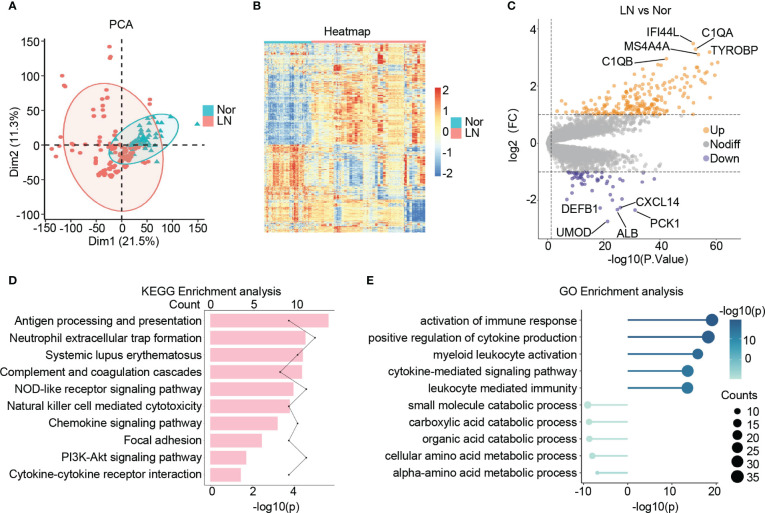
Identification of the DEGs in lupus nephritis. **(A)** The principal component analysis (PCA) showing the distribution of samples in patients with lupus nephritis and normal controls. **(B)** The heatmap illustrating the top 1000 genes with the highest standard deviation changes among individuals diagnosed with lupus nephritis and normal controls. **(C)** The volcano showing the expression of DEGs between lupus nephritis and normal controls. **(D)** The bar graph illustrating the significantly upregulated KEGG pathways in lupus nephritis compared to the normal controls. **(E)** The lollipop graph illustrating the upregulated(right) and downregulated(left) GO terms in lupus nephritis compared to the normal controls.

Following a principal component analysis (PCA) and hierarchical clustering analysis on the combined samples, a clear differentiation between patients with LN and normal controls is evident (**Figure 2A**; **Figure S1G**). Furthermore, the transcriptional profiles of patients with LN exhibit distinct characteristics compared to those of normal controls, indicating a unique status in LN (**Figure 2B**). Consequently, a differential expression analysis was conducted, leading to the identification of 270 significantly differentially expressed genes (DEGs) in LN. Out of these, 214 genes showed significant up-regulation, including C1QA, IFI44L, TYROBP, MS4A4A, and C1QB, which are known to be involved in immune cell activation and the inflammatory response in the glomerulus. Additionally, 56 genes displayed significant down-regulation, including ALB, UMOD, PCK1, CXCL14, and DEFB1, which are closely associated with the structure, function, and metabolism of the glomerulus (**Figure 2C**).

### Function enrichment analysis

Upon conducting KEGG analysis, we identified several enriched pathways in LN compared to normal controls. These pathways include antigen processing and presentation, neutrophil extracellular trap formation, systemic lupus erythematosus, complement and coagulation cascades, and NOD-like receptor signaling pathway. Furthermore, there was a noticeable upregulation of signaling pathways associated with cytokines and chemokines in LN (**Figure 2D**).

In terms of GO: BP enrichment analysis, we observed a significant up-regulation of immune responses, including the activation of immune response, myeloid leukocyte activation, and cytokine-mediated signaling pathway. Conversely, glomerular metabolism, particularly small molecule catabolic process, carboxylic acid catabolic process, organic acid catabolic process, and cellular amino acid metabolic process, showed significant down-regulation (**Figure 2E**).

Taking all these findings into consideration, it suggests an increase in the inflammatory immune microenvironment within the glomerulus in LN. This increase is characterized by elevated release of cytokines and chemokines, as well as increased infiltration of immune cells, predominantly myeloid cells. Additionally, we observed structural and metabolic damage in the glomerulus of patients with LN, observations consistent with previous studies and further validating our study’s convincing evidence (36–39).

### Construction of WGCNA network of LN

**Figure 3:**
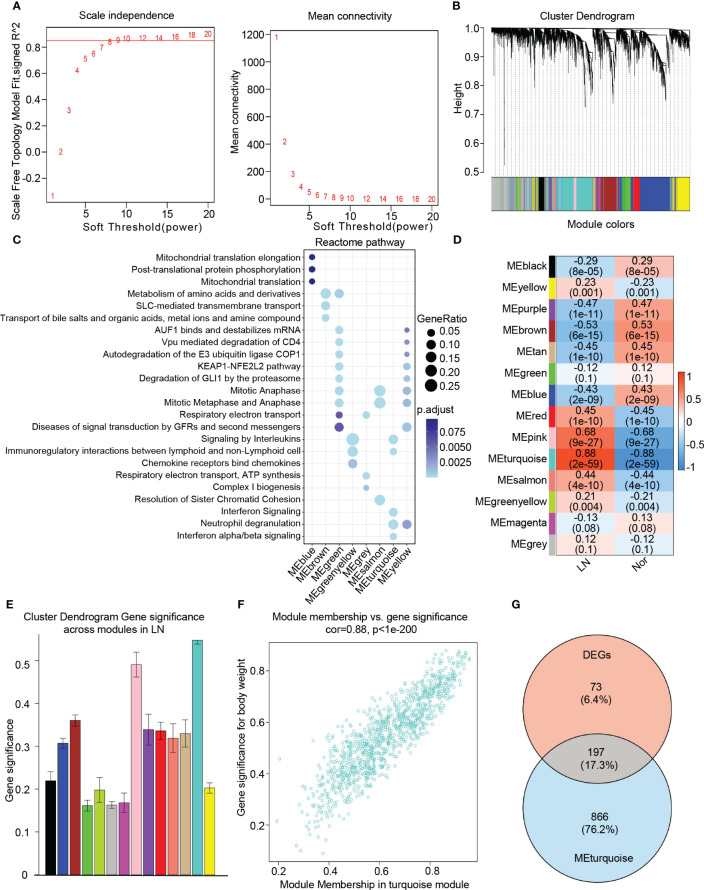
Identification of candidate hub genes based the WGCNA analysis. **(A)** The soft threshold power(left) and mean connectivity(right) of WGCNA network. **(B)** The cluster dendrogram of WGCNA network. **(C)** The dot plot showing the top enriched reactome pathways among different modules. **(D)** The heatmap depicting the relationship between the modules and clinical traits, specifically lupus nephritis and normal controls. **(E)** The bar chart illustrating the gene significance among different modules in lupus nephritis. **(F)** The scatter plot between gene significance (GS) and module members (MM) in turquoise module. **(G)** The venn diagram of the intersection of DEGs, turquoise module genes.

To identify key genes associated with the LN phenotype more precisely, we utilized WGCNA analysis on normal and LN samples. We set the soft threshold to 9 to ensure a scale-free topology of the network, as indicated by the results of the scale-free topology model fit and mean connectivity (**Figure 3A**). By assessing gene correlation, we constructed a gene hierarchy clustering dendrogram, which allowed us to identify 14 distinct gene modules exhibiting similar patterns of co-expression (**Figure 3B**).

To comprehensively investigate the function of each module, we performed a Reactome pathway enrichment analysis. The blue module was associated with mitochondrial translation, while the brown module demonstrated a relationship with diverse metabolic processes, including amino acid and metal ion metabolism. The green and yellow modules were primarily associated with cell cycle regulation, encompassing AUF1 binding, mRNA destabilization, mitotic anaphase, and metaphase. Furthermore, these modules showed enrichment in the KEAP1-NFE2L2 pathway, autodegradation of the E3 ubiquitin ligase COP1, and degradation of GLI1 via the proteasome. The greenyellow modules were found to participate in cytokine and chemokine-induced signaling pathways, specifically involving the binding of chemokines to interleukin and chemokine receptors. Additionally, these modules exhibited involvement in immunoregulatory interactions between lymphoid and non-lymphoid cells. On the other hand, the grey module was associated with respiratory electron transport, the salmon module was linked to mitosis, and the turquoise module was related to interferon signaling, neutrophil degranulation, as well as interferon alpha/beta signaling (**Figure 3C**).

To identify modules closely associated with LN, we performed correlation analysis between each module and phenotypes. Our analysis revealed that the “turquoise” module, consisting of 1063 genes, exhibited the highest clinical relevance to LN. This determination was based on a correlation coefficient of 0.88 and a p-value of 2e-59, indicating a strong correlation between module feature values and LN phenotypes (**Figures 3D, E**). Additionally, a significant correlation was observed between gene significance (GS) and module membership (MM) within the “turquoise” module, with a correlation coefficient of 0.88 and a p-value of 1e-200 (**Figure 3F**). Therefore, we identified the “turquoise” module as a key module associated with LN. Furthermore, the genes that appeared in both the DEGs and the turquoise module were considered as candidate hub genes in LN (**Figure 3G**). Additionally, based on our findings, we discovered that the turquoise module contains 1,063 genes, of which 197 overlap with DEGs, accounting for a significant proportion (approximately 73%) of DEGs. This validates the crucial role of the turquoise module in LN further, as other modules show lower overlap with DEGs. By filtering the overlapping genes, the turquoise module can more precisely identify genes closely associated with lupus nephritis progression.

### Identification and validation of hub genes in LN

**Figure 4:**
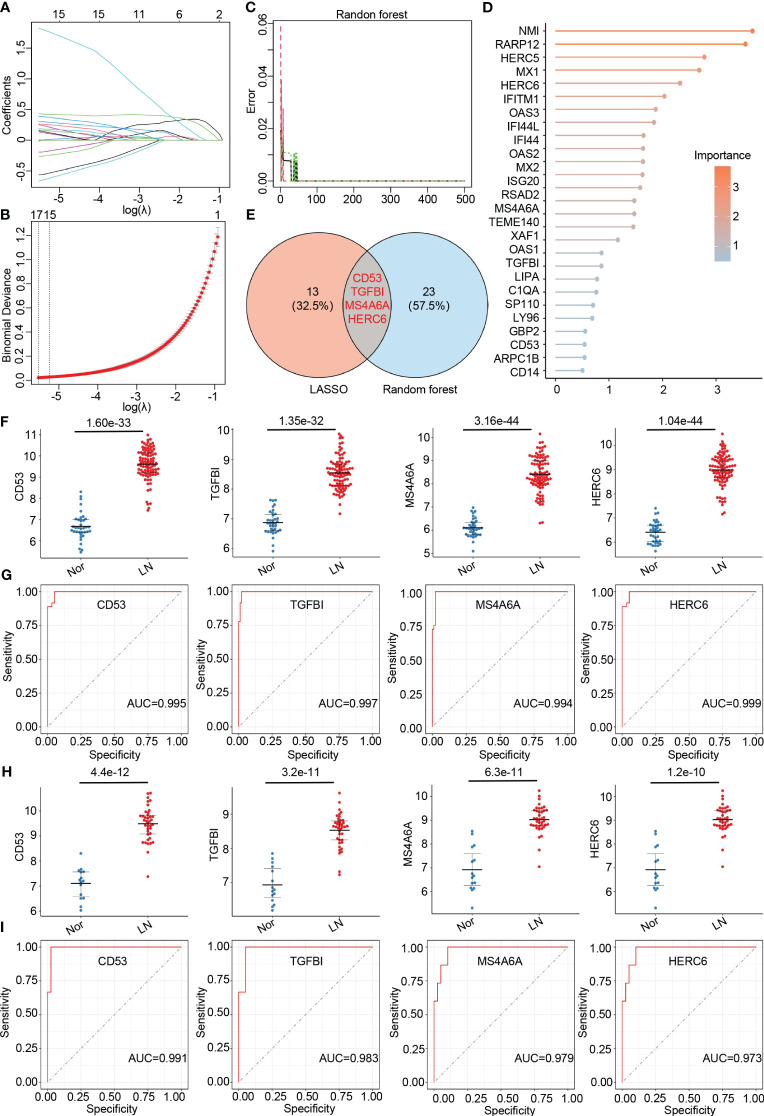
Identification of final hub genes by lasso regression analysis and random forest analysis. **(A)** Path diagram of the LASSO coefficients for the hub genes associated with lupus nephritis in training group. **(B)** LASSO regression cross-validation curve. Optimal λ values were determined using 10-fold cross-validation in training group. **(C)** The error rate confidence intervals for random forest mode in training group. **(D)** The lollipop graph illustrating the relative importance of genes in the random forest model within training group. **(E)** The venn diagram of the intersection of LASSO and random forest signature genes. **(F)** Expression levels of four hub genes in lupus nephritis patients compared with normal controls in training group. **(G)** ROC analysis of four hub genes in training group **(H)** Expression levels of four hub genes in lupus nephritis patients compared with normal controls in validation group. **(I)**. ROC analysis of four hub genes in validation group.

To further identify hub genes associated with LN, we applied two machine learning methods - LASSO regression and random forest - to the genes that overlap between the turquoise module and DEGs. LASSO analysis identified 17 hub genes (**Figures 4A, B**), while the random forest approach identified 27 hub genes (**Figures 4C, D**). By comparing the results, we found that the genes CD53, TGFBI, MS4A6A, and HERC6 were common to both methods and thus selected as the final hub genes associated with LN (**Figure 4E**).

To validate the accuracy of the final hub genes, we examined their expression levels in the training set. We observed that CD53, TGFBI, MS4A6A, and HERC6 were significantly upregulated in LN patients compared to the normal controls, suggesting their potential role in LN (**Figure 4F**). Furthermore, we calculated the area under the receiver operating characteristic curve (AUC-ROC) for each hub gene, resulting in values of 0.995 for CD53, 0.997 for TGFBI, 0.984 for MS4A6A, and 0.999 for HERC6 (**Figure 4G**). These AUC-ROC values indicate high diagnostic efficiency of the hub genes in predicting LN.

We also evaluated the diagnostic efficiency of these hub genes in the validation group. Consistent with the training group, these hub genes exhibited higher expression levels in LN patients (**Figure 4H**). The AUC-ROC values in the validation group were 0.991 for CD53, 0.983 for TGFBI, 0.979 for MS4A6A, and 0.973 for HERC6, respectively (**Figure 4I**). These findings further support the remarkable diagnostic efficiency of the identified hub genes in predicting LN.

### GSEA analysis of hub genes

**Figure 5:**
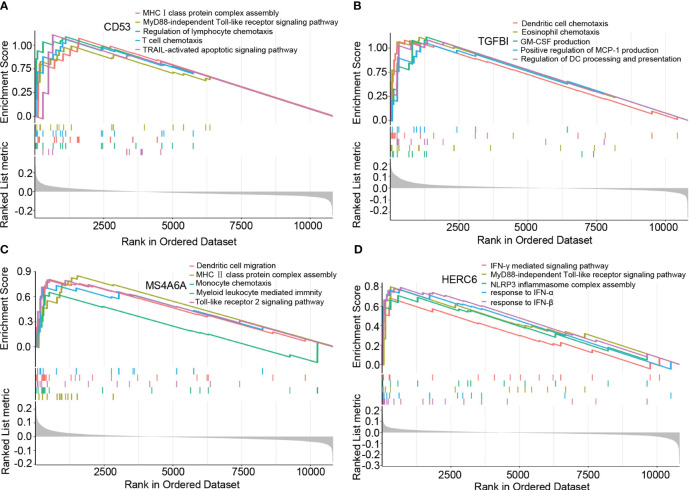
The GSEA of hub genes in lupus nephritis. **(A)** The GSEA of CD53 in lupus nephritis. **(B)** The GSEA of TGFBI in lupus nephritis. **(C)** The GSEA of MS4A6A in lupus nephritis. **(D)** The GSEA of HERC6 in lupus nephritis.

Based on the results of the GSEA analysis, we identified significant correlations between the hub genes and various signaling pathways associated with LN. CD53 showed significant correlations with pathways such as MHCI class protein complex assembly, MyD88-independent Toll-like receptor signaling pathway, regulation of lymphocyte chemotaxis, T cell chemotaxis, and TRAIL-activated apoptotic signaling pathway (**Figure 5A**). TGFBI exhibited significant correlations with pathways such as dendritic cell chemotaxis, eosinophil chemotaxis, GM-CSF production, positive regulation of MCP-1 production, and regulation of dendritic cell processing and presentation (**Figure 5B**). MS4A6A showed significant correlations with pathways such as dendritic cell chemotaxis, MHCII class protein complex assembly, monocyte chemotaxis, myeloid leukocyte-mediated immunity, and Toll-like receptor 2 signaling pathway (**Figure 5C**). Lastly, HERC6 displayed significant correlations with pathways such as IFN-γ mediated signaling pathway, MyD88-independent Toll-like receptor signaling pathway, NLRP3 inflammasome complex assembly, response to IFN-α, and response to IFN-β (**Figure 5D**).

These findings provide valuable insights into the potential mechanisms underlying LN, as the identified hub genes are involved in distinct signaling pathways associated with the disease.

### Analysis of immune infiltration in LN

**Figure 6:**
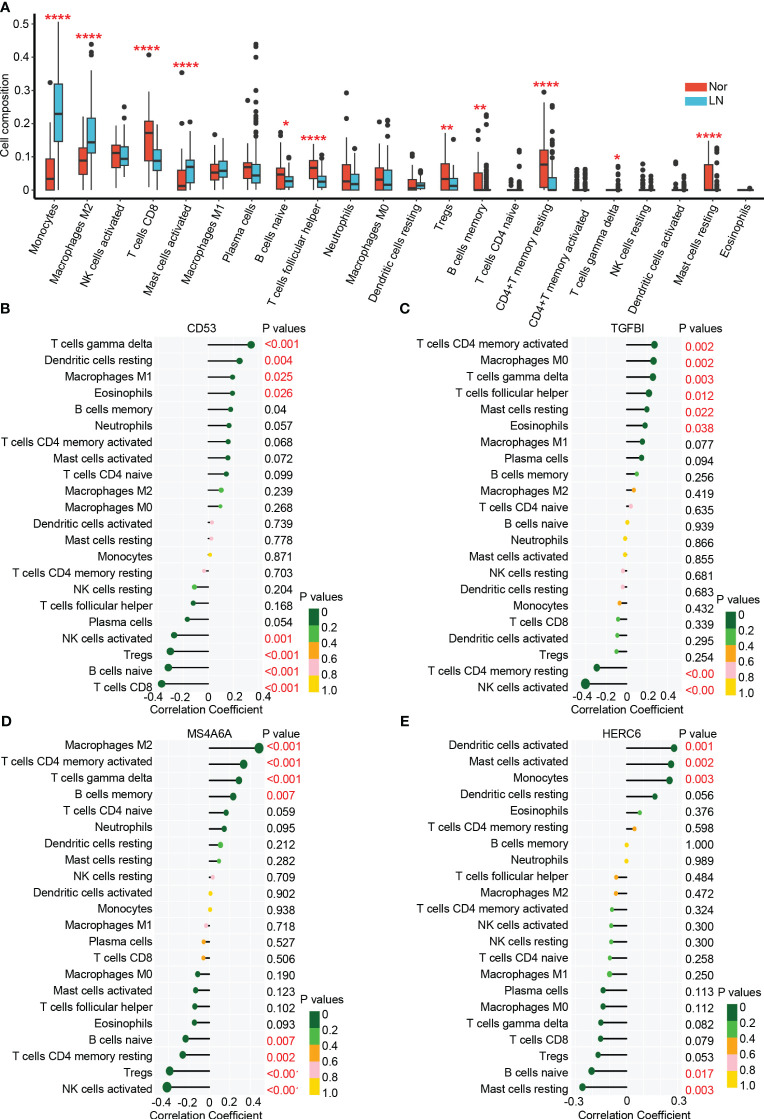
The immune cell infiltration association with hub genes. **(A)** The immune cell infiltration between lupus nehritis and normal controls. **(B)** The association between CD53 and different immune cell infiltration in lupus nephritis. **(C)** The association between TGFBI and different immune cell infiltration in lupus nephritis. **(D)** The association between MA4A6A and different immune cell infiltration in lupus nephritis. **(E)** The association between HERC6 and different immune cell infiltration in lupus nephritis. * p < 0.05;** P < 0.01;*** P < 0.0001.

In the glomerulus of the kidneys, the differential analysis revealed significant differences in immune cell infiltration between patients with LN and normal controls. Specifically, LN patients showed a significant increase in infiltrating monocytes (p < 0.001), macrophages M2 (p < 0.001), activated mast cells (p < 0.001), memory B cells (p < 0.01), and γδT cells (p < 0.05) (**Figure 6A**). On the other hand, CD8+ T cells (p < 0.001), naive B cells (p < 0.05), follicular helper T cells (p < 0.001), regulatory T cells (p < 0.01), and resting memory CD4+ T cells (p < 0.001) were significantly higher in normal controls. These results suggest that myeloid cells including monocytes and macrophages M2 are the main infiltrating immune cells in the glomerulus of LN affected kidneys. These cells may significantly contribute to LN disease pathogenesis (22, 40, 41).

### Correlation of hub genes with infiltrating immune cells in LN

The analysis of immune cell infiltration in the glomerulus of the kidneys revealed interesting associations between the hub genes (CD53, TGFBI, MS4A6A, and HERC6) and specific immune cell types.

CD53 exhibited positive correlations with the infiltration of γδT cells, resting dendritic cells, M1 macrophages, and eosinophils, while displaying negative correlations with the infiltration of activated NK cells, T NK cells, Tregs, naive B cells, and CD8 T cells (**Figure 6B**). Similarly, TGFBI showed positive correlations with the infiltration of activated memory CD4 T cells, M0 macrophages, γδT cells, follicular helper T cells, resting mast cells, and eosinophils. However, it displayed a negative correlation with the infiltration of resting memory CD4 T cells and activated NK cells (**Figure 6C**). Moreover, MS4A6A exhibited positive correlations with the infiltration of M2 macrophages, activated memory CD4 T cells, γδT cells, and memory B cells, while showing negative correlations with the infiltration of naive B cells, resting memory CD4 T cells, Tregs, and activated natural killer cells (**Figure 6D**). Lastly, HERC6 displayed positive correlations with the infiltration of activated dendritic cells, activated mast cells, and monocytes, but showed a negative correlation with the infiltration of naive B cells and resting mast cells (**Figure 6E**).

Altogether, based on the functionality of genes (**Figure 5**) and the correlation between immune infiltration and gene expression (**Figure 6**), we have reached the following conclusions: CD53 primarily facilitates the infiltration of γδ T cells by engaging in MHC class I antigen presentation (42, 43). TGFBI plays a significant role in the infiltration of mast cells and eosinophils. MS4A6A is involved in the infiltration of M2 macrophages in the glomerulus through the TLR2 signaling pathway and MHC class II antigen presentation (44–46).While, HERC6, which operates via the MyD88-dependent Toll-like receptor signaling pathway, broadly mediates the infiltration of myeloid-derived immune cells, including monocytes and mast cells (47).

These findings shed light on the associations between the hub genes and specific immune cell infiltrations in the glomerulus of the kidneys in LN. The identified correlations provide valuable insights into the potential roles of these genes in the immune response and pathogenesis of LN.

### The relationships between hub genes and clinical and pathological features in LN

The prognosis of LN is influenced by factors such as pathologic classification, renal function, and proteinuria levels. To investigate the connections between hub genes and clinical as well as pathological features, an analysis was conducted using the Nephroseq database.

**Figure 7:**
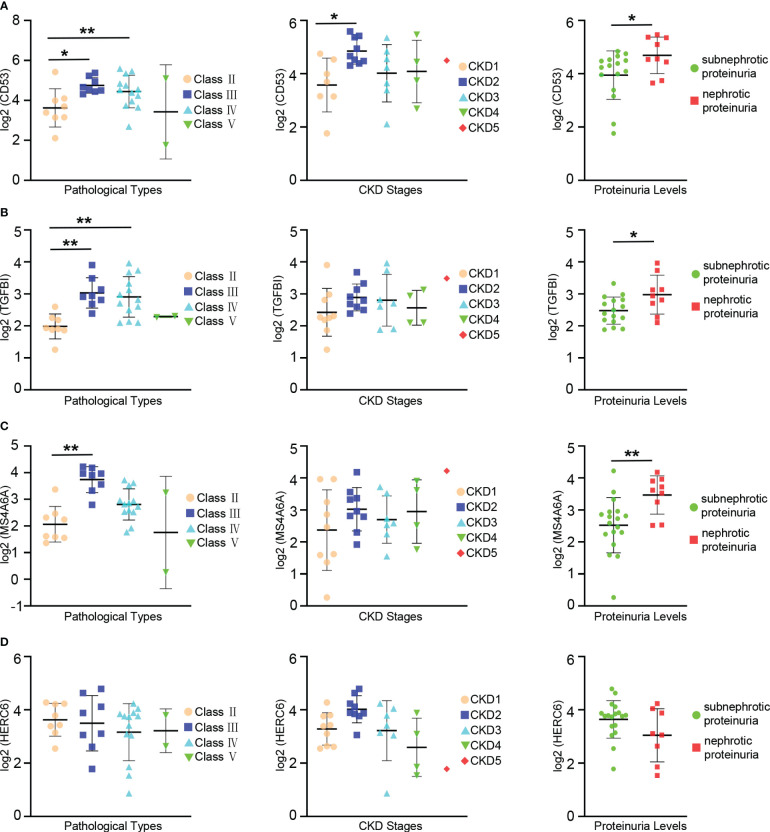
Relationships between the expression of hub gene and pathological classification, stage of chronic kidney disease(CKD), and proteinuria. **(A)** The scatter plots depicting the relationship between the expression level of the CD53 and three variables: pathological classification (eft), stage of CDK(center), and proteinuria (right). **(B)** The scatter plots depicting the relationship between the expression level of the TGFBI and three variables: pathological classification (left), stage of CKD (center), and proteinuria (right). **(C)** The scatter plots depicting the relationship between the expression level of the MS4A6A and three variables: pathological classification (left), stage of CKD (center), and proteinuria (right). **(D)** The scatter plots depicting the relationship between the expression level of the HERC6 and three variables: pathological classification (left), stage of CKD (center), and proteinuria (right). * p < 0.05;** P < 0.01.

The expression levels of CD53 were notably higher in patients with class III and IV LN compared to those with class II. Similar trends were observed when comparing patients with CKD stage 2 and stage 1, where CD53 expression levels were significantly elevated. Although CD53 expression increased in patients with CKD stage 3 and 4 compared to stage 1, the difference did not reach statistical significance. Furthermore, a correlation was found between CD53 expression and proteinuria, with significantly higher levels in patients experiencing nephrotic proteinuria compared to those with subnephrotic proteinuria (**Figure 7A**).

A significant increase in TGFBI expression levels was observed in patients with class III and IV LN compared to those with class II. However, despite relatively high CD53 expression in patients with CKD stages 2, 3, and 4 compared to stage 1, the difference did not reach statistical significance. Additionally, there was a correlation between TGFBI expression and proteinuria, with significantly higher levels in patients with nephrotic proteinuria compared to those with subnephrotic proteinuria (**Figure 7B**).

Similarly, the expression levels of MS4A6A were significantly elevated in patients with class III lupus nephritis compared to those with class II. However, despite observing higher MS4A6A expression in patients with CKD stages 2, 3, and 4 compared to stage 1, the difference did not reach statistical significance. There was an association between MS4A6A expression and proteinuria, with notably higher levels in patients with nephrotic proteinuria compared to those with subnephrotic proteinuria (**Figure 7C**). Conversely, no linear relationship was found between HERC6 expression and pathological classification, stages of CKD, or proteinuria (**Figure 7D**).

Overall, these hub genes are strongly associated with the prognosis of LN.

### The distribution and expression of hub genes in kidney

**Figure 8:**
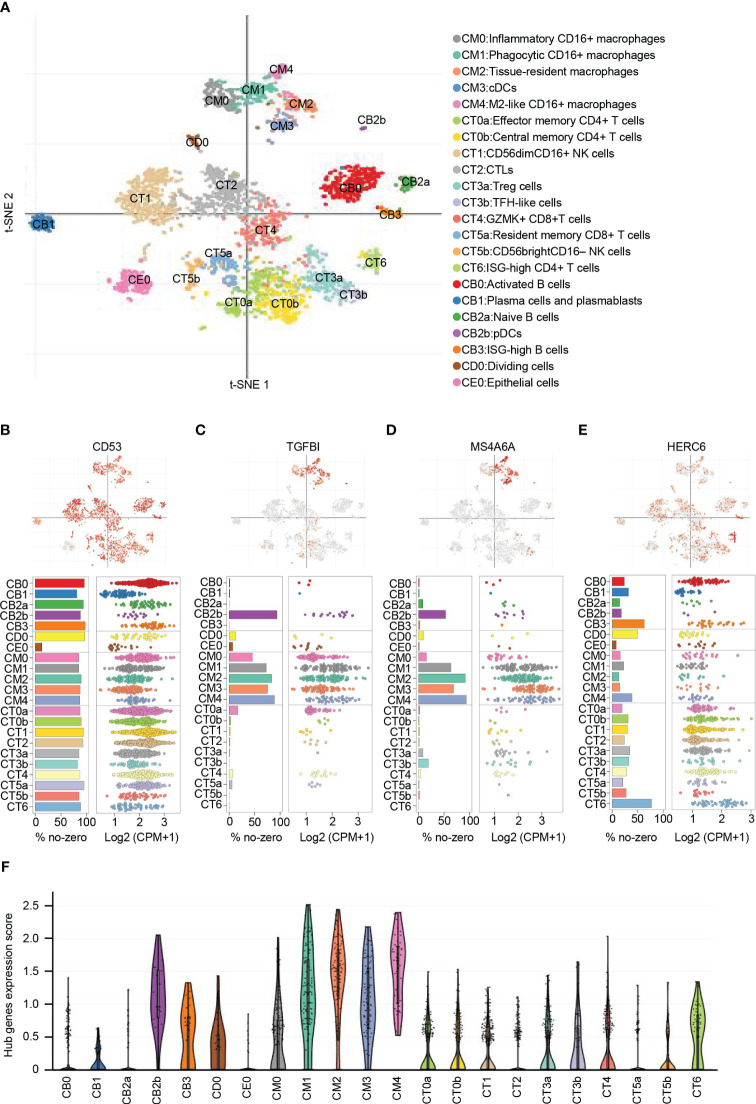
Distribution and expression of hub genes based on the single-cell RNA sequencing data. **(A)** t-SNE plot showing the 22 identified cell clusters. **(B)** Featureplot, bar plot and dot plot showing the distribution and expression of CD53. **(C)** Featureplot, bar plot and dot plot showing the distribution and expression of TGFBI. **(D)** Featureplot, bar plot and dot plot showing the distribution and expression of MS4A6A. **(E)** Featureplot, bar plot and dot plot showing the distribution and expression of HERC6. **(F)** Violin plots showing combined expression scores of hub genes.

From the publicly available single-cell RNA sequencing (scRNA-seq) data of LN, a total of 21 immune cell clusters were identified (22). These clusters primarily consisted of macrophages, dendritic cells (DCs), T cells, natural killer (NK) cells, and B cells. Additionally, there was a single cluster of epithelial cells that was identified as well (**Figure 8A**).

CD53 was found to be widely distributed in immune cells. In contrast, TGFBI was predominantly expressed in various macrophage subsets, including inflammatory CD16+ macrophages (CM0), phagocytic CD16+ macrophages (CM1), tissue-resident macrophages (CM2), conventional dendritic cells (CM3), M2-like CD16+ macrophages (CM4), and plasmacytoid dendritic cells (CB2b). Similarly, MS4A6A showed primary expression in CM1, CM2, CM3, CM4, and CB2b. On the other hand, HERC6 exhibited primary expression in ISG-high CD4+ T cells (CT6) and ISG-high B cells (CB3), with lower expression observed in other types of T cells and NK cells (**Figures 8B-E**).

To analyze the distribution of the gene signature represented by the hub genes, we calculated the average expression levels of these genes. The results indicated that CM0, CM1, CM2, CM3, CM4, and CB2b exhibited the highest scores, suggesting the importance of these cell types in LN (**Figure 8F**). These findings emphasize the potential significant role of macrophages, dendritic cells, and ISG-high lymphocytes in LN.

## Discussion

The study employed comprehensive bioinformatics and machine learning techniques to identify four hub genes: CD53, TGFBI, MS4A6A, and HERC6. These genes were found to be upregulated in LN and played a significant role in mediating the inflammatory response and immune activation. The findings also indicated a strong association between these genes and immune cell infiltration, particularly in macrophages, monocytes, and γδT cells. Moreover, the study revealed a significant correlation between these genes and various clinicopathological features of LN, especially in terms of classification, renal function, and proteinuria, suggesting their potential involvement in the prognosis of LN. Additionally, macrophages and DCs exhibited the highest hub gene expression scores, further implicating these four hub genes in the development of LN. Consequently, these genes have the potential to serve as novel candidate driver genes.

The advancement of gene microarray technology and high-throughput techniques has made bioinformatics methods crucial for efficiently identifying differentially expressed genes (DEGs) in LN (22, 48). In our study, we identified the top five up-regulated DEGs in LN as C1QA, IFI44L, TYROBP, MS4A4A, and C1QB. C1QA and C1QB are components of the classical complement system, which plays a critical role in activating immune responses, promoting inflammatory reactions, and facilitating immune cell infiltration (49–52). These genes are extensively expressed in macrophages, indicating the potential significant role of macrophages in LN (53, 54). IFI44L promoter methylation has been reported as a potential blood biomarker for SLE and has been identified and validated as a biomarker for LN (55–57). TYROBP encodes a transmembrane signaling polypeptide that binds to NK cell activity receptors and activates signal transduction (58, 59). Previous studies have shown a strong correlation between TYROBP and proteinuria in SLE (60). On the other hand, MS4A4A is expressed during the differentiation of monocyte-macrophage cells and is upregulated by M2-like signals, including IL-4 and dexamethasone (61–64). Although the exact mechanism of MS4A4A’s upregulation in LN remains unclear, our research findings confidently confirm the significant roles played by the complement system, interferon signaling pathway, and immune cell infiltration in LN.

Using a combination of bioinformatics techniques and sophisticated machine learning methods, we have successfully identified four hub genes: CD53, TGFBI, MS4A6A, and HERC6. CD53, also known as OX44 or TSPAN25, is an exclusive tetraspanin protein expressed in the immune system. It is found on the surface of B cells, CD4+ T cells, CD8+ T cells, DCs, macrophages, and NK cells, suggesting a broader role within the immune system (65–67). CD53 interacts with integrins and other adhesion molecules interacts with integrins and other adhesion molecules, particularly LFA-1, on NK cells and various types of immune cells, enhancing the adhesion, migration, and proliferation of immune cells (68–70). Additionally, CD53 acts as a regulator of immune cell signaling. Recent studies have identified CD2, IL-7R, MHC-I, and MHC-II proteins as partners of CD53, demonstrating its role in modulating downstream intracellular signaling pathways (71–74). Our research revealed an upregulation of CD53 expression in LN and its widespread presence in immune cells, highlighting its crucial role in regulating the migration of immune cells, including γδT cells, DCs, and macrophages, and promoting immune activation. Furthermore, a comprehensive analysis of the clinical implications of CD53 shows a positive correlation between its expression and proteinuria, a negative correlation with renal function, and an association with adverse renal pathology. In conclusion, our findings strongly suggest that CD53 may contribute to the exacerbation of LN by orchestrating immune cell migration and promoting immune activation.

TGFBI, a protein that is produced in response to transforming growth factor beta (TGF-β), has been shown to have significant implications in various kidney diseases (75–77). It can be detected in both serum and urine, making it accessible for diagnostic purposes (76). In our research, we observed an upregulation of TGFBI expression in LN, particularly in macrophages, cDCs, and pDCs, indicating its crucial role in these cell types. Moreover, we have observed that TGFBI plays an essential role in regulating DC migration and eosinophil chemotaxis, which further suggests its involvement in the diagnosis and pathogenesis of LN. Furthermore, a comprehensive analysis of the clinical relevance of TGFBI has revealed significant associations. Higher TGFBI expression showed a positive correlation with proteinuria but a negative correlation with renal function. Additionally, increased levels of TGFBI expression were associated with more severe pathological features. These findings provide a solid foundation for future research and development in this field, with the aim to utilize TGFBI as a diagnostic tool and explore its therapeutic potential

MS4A6A encodes a member of the membrane-spanning 4A gene family, which exhibits distinct expression patterns among hematopoietic cells and nonlymphoid tissues (78, 79). Previous studies have reported associations between MS4A6A and the development and progression of neurodegenerative diseases, including Alzheimer’s and Parkinson’s (80–82). Furthermore, MS4A6A is also associated with kidney disease. In the context of impaired kidney transplant outcome, complement-activating anti-HLA donor-specific antibodies (DSAs) have been linked to MS4A6A, as they are highly associated with circulating complement-activating anti-HLA DSAs (83). In our research, we have confirmed an upregulation of MS4A6A expression in LN. Specifically, MS4A6A exhibits predominant expression in macrophages and DCs, and plays a role in the migration of myeloid leukocytes and myeloid leukocyte-mediated immunity. Analysis of immune infiltration has revealed a robust correlation between MS4A6A expression and macrophage infiltration. Additionally, a comprehensive analysis of the clinical relevance of MS4A6A has shown a positive correlation between MS4A6A expression and proteinuria. Moreover, elevated levels of MS4A6A expression were found to be linked to more severe pathological features. However, the precise pathogenic mechanism remains unclear and warrants further investigation. It is speculated that the high expression of MS4A6A in macrophages and DCs may facilitate the migration of immune cells, contributing to the augmentation of the inflammatory immune microenvironment at the site of injury in LN.

HERC proteins belong to the HECT family and serve as ubiquitin E3 ligases. The HERC family of ubiquitin ligases plays a crucial role in various essential cellular processes, including neurodevelopment, DNA damage response, cell proliferation, cell migration, and immune responses. HERC6 is a member of the HECT family (84, 85). Several studies focusing on LN have observed elevated expression levels of HERC6 (57, 86, 87). Our research findings support the notion that HERC6 expression is increased in individuals with LN. Specifically, HERC6 shows predominant expression in ISG-high CD4+ T cells and ISG-high B cells, where it plays a role in IFN-related signaling pathways such as IFN-α/β/γ, as evidenced by GSEA analysis. Numerous studies have demonstrated the critical involvement of the IFN signaling pathway in the development and progression of LN (21, 37, 88–90). However, our correlation analysis between HERC6 and clinical information did not reveal any significant correlations. This lack of correlation may be attributed to the complex functionality of HERC6, suggesting that its relationship is not simply linear. In terms of immune infiltration analysis, HERC6 exhibits a positive correlation with the infiltration of activated DCs, activated mast cells, and monocytes. Our hypothesis posits that the overexpression of HERC6 in ISG-high immune cells initiates inflammation and immune responses via the interferon signaling pathway. This subsequently results in the infiltration of myeloid-derived immune cells, specifically monocytes, macrophages, and dendritic cells, into the renal glomeruli, thereby exacerbating LN.

In LN, immune cell infiltration, particularly monocytes and macrophages, plays a crucial role in disease progression by accumulating in the kidney and promoting inflammation through the production of chemokines and cytokines (40, 41, 91–94). Additionally, LN patients’ pDCs produce type I interferons, which can activate B cells and lead to the production of autoreactive antibodies (95–97). Although the presence of other lymphocytes in LN kidney biopsies, such as innate lymphoid cells and γδT cells, has been observed, their exact contribution to the disease remains unclear (98–102). In our study, we observed a significant increase in monocytes and M2 macrophages as the main infiltrating immune cells in the glomeruli of patients with LN. Additionally, we found a significant increase in mast cells and γδT cells, as well as a decrease in Tregs. Furthermore, the hub gene was predominantly expressed in the aforementioned major infiltrating immune cells, highlighting the crucial role of the inflammatory immune microenvironment in the development of LN. However, contrary to previous research findings, we observed a decrease in CD8+ T cells in LN. Further investigation is required to determine the underlying cause of this phenomenon.

Our research has successfully identified 270 DEGs, providing valuable insights into the specific transcriptional profile of LN. During our investigation, we focused specifically on four upregulated hub genes: CD53, TGFBI, MS4A6A, and HERC6 in LN. These genes have significant roles in regulating the chemotaxis of monocytes and macrophages, orchestrating interferon signaling pathways, and activating inflammatory responses. Importantly, they are closely associated with adverse outcomes in LN. The expression of CD53, TGFBI, MS4A6A, and HERC6 in macrophages, DCs, and ISG-high lymphocytes correlates with the known immune cell infiltration patterns observed in LN. Based on this observation, we propose that CD53+ immune cells, TGFBI+ or MS4A6A+ monocytes and macrophages, and HERC6+ ISG-high lymphocytes may play a crucial role in the development of glomerular lesions in LN. Consequently, these hub genes and immune cell populations have the potential to be targeted for immunotherapy in LN patients, opening up new avenues for therapeutic interventions. Further studies are needed to validate these hypotheses and explore their clinical applications.

## Data availability statement

The datasets presented in this study can be found in online repositories. The names of the repository/repositories and accession number(s) can be found in the article/**Supplementary Material**.

## Author contributions

ZW: Conceptualization, Data curation, Formal analysis, Methodology, Software, Visualization, Writing – original draft, Writing – review & editing. DH: Conceptualization, Methodology, Software, Writing – original draft. GP: Conceptualization, Funding acquisition, Writing – original draft. RZ: Conceptualization, Funding acquisition, Investigation, Supervision, Writing – review & editing. YY: Funding acquisition, Investigation, Supervision, Writing – review & editing.

## Supplementary material

Supplementary Figure 1 Distribution and expression of datasets of lupus nephritis from GEO. **(A)** Box plot showing the distribution and expression of GSE99399. **(B)** Box plot showing the distribution and expression of GSE104948. **(C)** Box plot showing the distribution and expression of GSE32591. **(D)** Box plot showing the distribution and expression of GSE127797. **(E)** Box plot showing the distribution and expression of merged data before batch removal. **(F)** Box plot showing the distribution and expression of merged data after batch removal. **(G)** Hierarchical clustering showing the distribution of patients with lupus and normal controls.
