# Supplementary figures and images for: Identification of driver genes in lupus nephritis based on comprehensive bioinformatics and machine learning
Source: Front Immunol. 2023 Dec 7;14:1288699. doi: 10.3389/fimmu.2023.1288699 (PMC10733527; doi:10.3389/fimmu.2023.1288699)

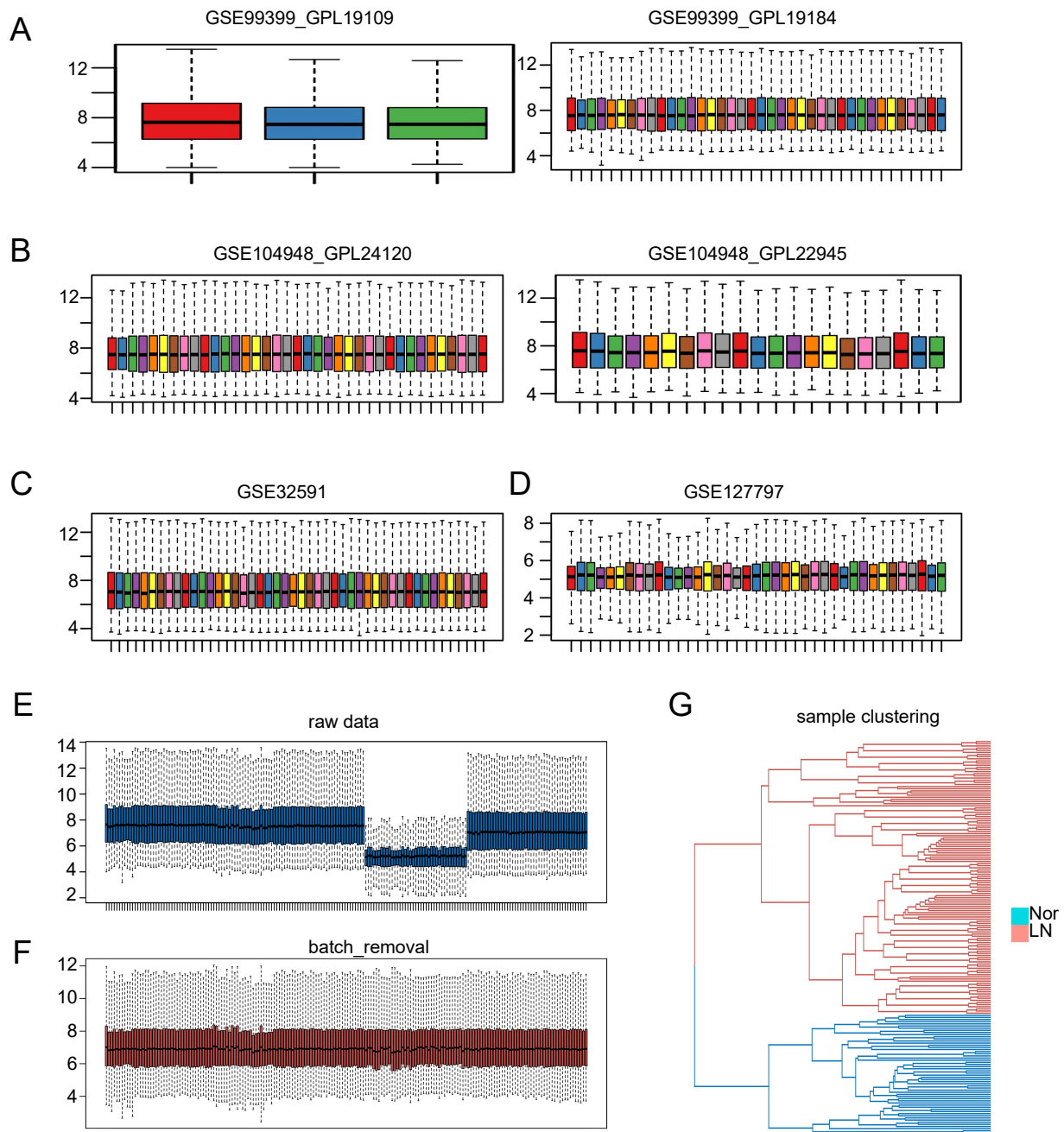

Supplement: Supplementary Figure 1 — Distribution and expression of datasets of lupus nephritis from GEO. (A) Box plot showing the distribution and expression of GSE99399. (B) Box plot showing the distribution and expression of GSE104948. (C) Box plot showing the distribution and expression of GSE32591. (D) Box plot showing the distribution and expression of GSE127797. (E) Box plot showing the distribution and expression of merged data before batch removal. (F) Box plot showing the distribution and expression of merged data after batch removal. (G) Hierarchical clustering showing the distribution of patients with lupus and normal controls. [file DataSheet_1.pdf]
